# A Case Report on Acute Pancreatitis in a Patient With Coronavirus Disease 2019 (COVID-19) Pneumonia

**DOI:** 10.7759/cureus.14628

**Published:** 2021-04-22

**Authors:** Aviral Gupta, Dharam P Bansal, Puneet Rijhwani, Vipasha Singh

**Affiliations:** 1 Department of General Medicine, Mahatma Gandhi Medical College and Hospital, Jaipur, IND; 2 Department of Radiology, Mahatma Gandhi Medical College and Hospital, Jaipur, IND

**Keywords:** covid-19, sars-cov-2, acute pancreatitis, viral pancreatitis, amylase, lipase, coronavirus disease 2019

## Abstract

With the ongoing coronavirus disease 2019 (COVID-19) pandemic, there has been an explosion of scientific literature on the clinical manifestations and pathogenesis of severe acute respiratory syndrome coronavirus 2 (SARS-CoV-2) infection. Gastrointestinal symptoms occur in 15-20% of COVID-19 patients; however, there have not been many case reports on acute pancreatitis in COVID-19 patients. The expression of ACE-2 ([angiotensin-converting enzyme 2] the host receptor for SARS-CoV-2) is very high in the pancreas, which might be a contributing factor, but the high expression is mainly localized to endocrine pancreas. This case report describes a case of a 25-year-old Indian female with COVID-19 with acute pancreatitis in the absence of any other known risk factors for pancreatitis.

## Introduction

The identification of a novel coronavirus had led to a cluster of severe pneumonia cases in Wuhan, China, in December 2019. The disease was later designated as coronavirus disease 2019 (COVID-19) [1] by the World Health Organization on March 11, 2020 [2]. Since its inception, there have been more than 83.3 million cumulative cases and 1.8 million deaths worldwide as of January 5, 2021 [3]. The clinical spectrum of the disease ranges from asymptomatic infection [4] to critical life-threatening respiratory disease [5]. However, the organ involvement in COVID-19 is not limited to the respiratory system, and several other organ systems can be involved. Data analysis indicates the presence of six distinct clusters of symptoms of COVID-19 [6], including gastrointestinal symptoms of varying severity. There has been evidence of pancreatic injury in COVID-19 patients, but this is often overlooked and the real prevalence of this complication is unknown. This case report highlights the case of a patient with COVID-19 pneumonia who developed acute pancreatitis.

## Case presentation

A 25-year-old Indian female with no significant past history presented to our emergency department with complaints of fever, headache, sore throat, dry cough, and loss of taste sensation for nine days, and abdominal pain with vomiting for two days. Her COVID reverse transcription polymerase chain reaction (RT-PCR) test performed on the second day of her illness returned positive. Subsequent to her first positive RT-PCR, the patient was started on oral favipiravir, tablet ivermectin 12 mg once daily for three days, and other supportive therapy. The patient did not have shortness of breath at this time or a history of smoking or alcohol intake. On the eighth day of illness, the patient started experiencing severe, dull aching, epigastric pain radiating to the back, with vomiting. The investigations on her eighth day of illness revealed that her repeat COVID RT-PCR was positive, C-reactive protein was 18.62 mg/L, D-dimer was highly elevated (>5,000 ng/mL) and serum lipase was 2,052.61 U/L (>3 times upper limit of normal). She was then admitted to our hospital on the ninth day of illness as a case of COVID-19 with suspected acute pancreatitis. On physical examination, her vitals were normal and epigastric tenderness was appreciated. Her investigations before and after admission are shown in Table 1, indicating a pro-inflammatory and hyper-coagulable state of COVID-19.

**Table 1:** Laboratory investigations of the patient on days of illness. ALP, alkaline phosphatase; COVID, coronavirus disease 2019; CRP, C-reactive protein; HIV, human immunodeficiency virus; ESR, erythrocyte sedimentation rate; HBsAg, hepatitis B surface antigen; HbA1c, hemoglobin A1c; HCV, hepatitis C virus; IL-6, interleukin-6; NLR, neutrophil-to-lymphocyte ratio; NT-proBNP, N-terminal-pro-B-type natriuretic peptide; PCR, reverse transcription polymerase chain reaction; SGOT, serum glutamic oxaloacetic transaminase; SGPT, serum glutamic pyruvic transaminase; TLC, total leucocyte count; TSH, thyroid-stimulating hormone; VDRL, Venereal Disease Research Laboratory test

Parameter	Day 9	Day 11	Day 13	Day 14	Day 15	Day 16
COVID RT-PCR	-	-	Negative	Negative	-	-
TLC (cells/mm^3^)	9700	7010	6730	7400	5490	6400
Neutrophil (cells/mm^3^)	7700	5570	5320	5700	4350	4780
Lymphocyte (cells/mm^3^)	1300	940	1110	1200	870	1000
NLR	5.7	5.92	4.79	4.9	5.0	4.78
Hemoglobin (g/dL)	11.6	10.0	10.0	10.1	11.0	11.2
Platelet count (x10^3^/mm^3^)	190	184	183	236	267	256
Eosinophil (cells/mm^3^)	100	80	0	-	0	90
CRP (mg/L)	52.6	81.9	62.1	34.9	27.6	37.4
D-dimer (ng/mL)	3627	11,100	1850	3600	442	606
NT-proBNP (pg/mL)	-	3990	2320	5010	-	-
Troponin I	-	0.021	<0.010	<0.010	-	-
Procalcitonin	0.030	0.24	0.13	0.096	-	-
Serum amylase	1814	-			-	-
Serum lipase	11920	-	301	275.3	225	327
IL-6	514.6	-			-	-
Urea	22.6	19.7	38	19.2	-	-
Creatinine	0.8	0.7	0.5	0.6	-	-
Calcium	9.2	8.7	9.0	-	-	-
Blood sugar	83.8	81.1	-	-	-	-
ESR	55	-	-	-	-	-
HbA1c	5.83	-	-	-	-	-
SGOT	24	23.4	-	-	-	-
SGPT	25.4	18.7	-	-	-	-
ALP	121.1	100.0	-	-	-	-
Serum bilirubin	0.6	0.8	-	-	-	-
Ferritin	44.9	-	-	-	-	-
Triglycerides	97.4	-	-	-	-	-
Prothrombin time	-	16.2	-	-	-	-
VDRL	Negative					
Malaria antigen	Negative					
HIV	Negative					
HBsAg	Negative					
Anti-HCV antibody	Negative					
Blood culture	Negative					
TSH	2.54					

**Figure 1:**
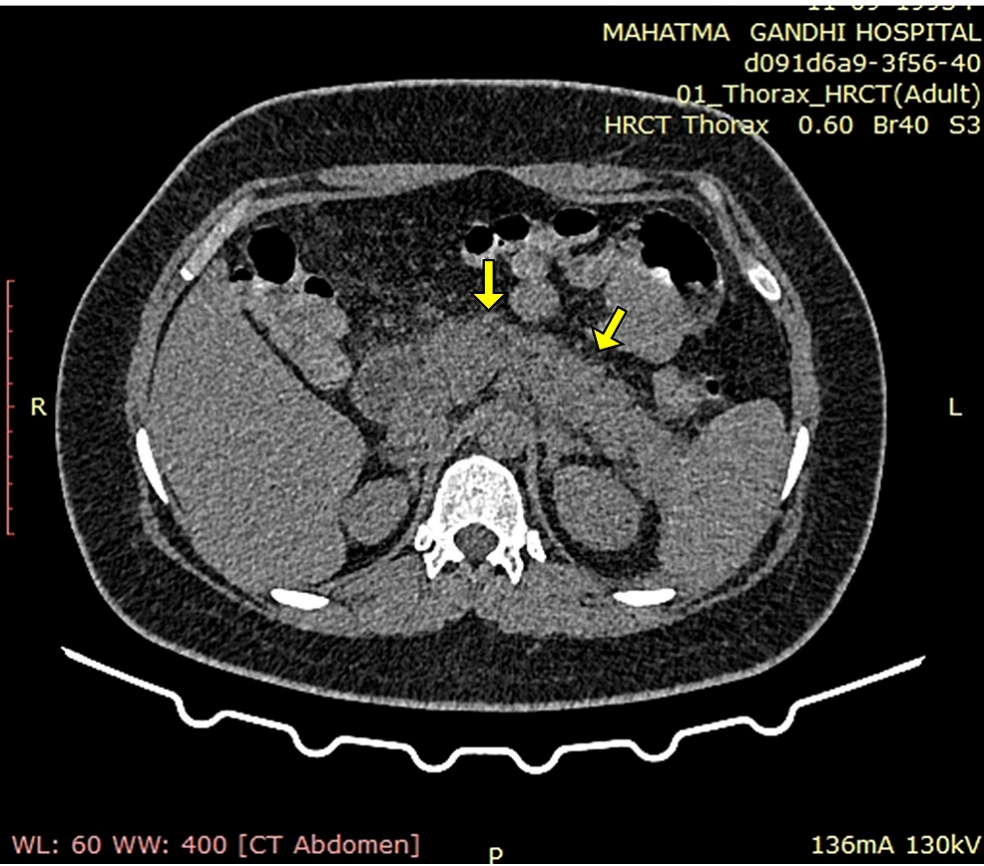
Non-contrast CT of the abdomen (axial section) showing diffusely bulky pancreas with no areas of calcification noted (solid yellow arrows).

**Figure 2:**
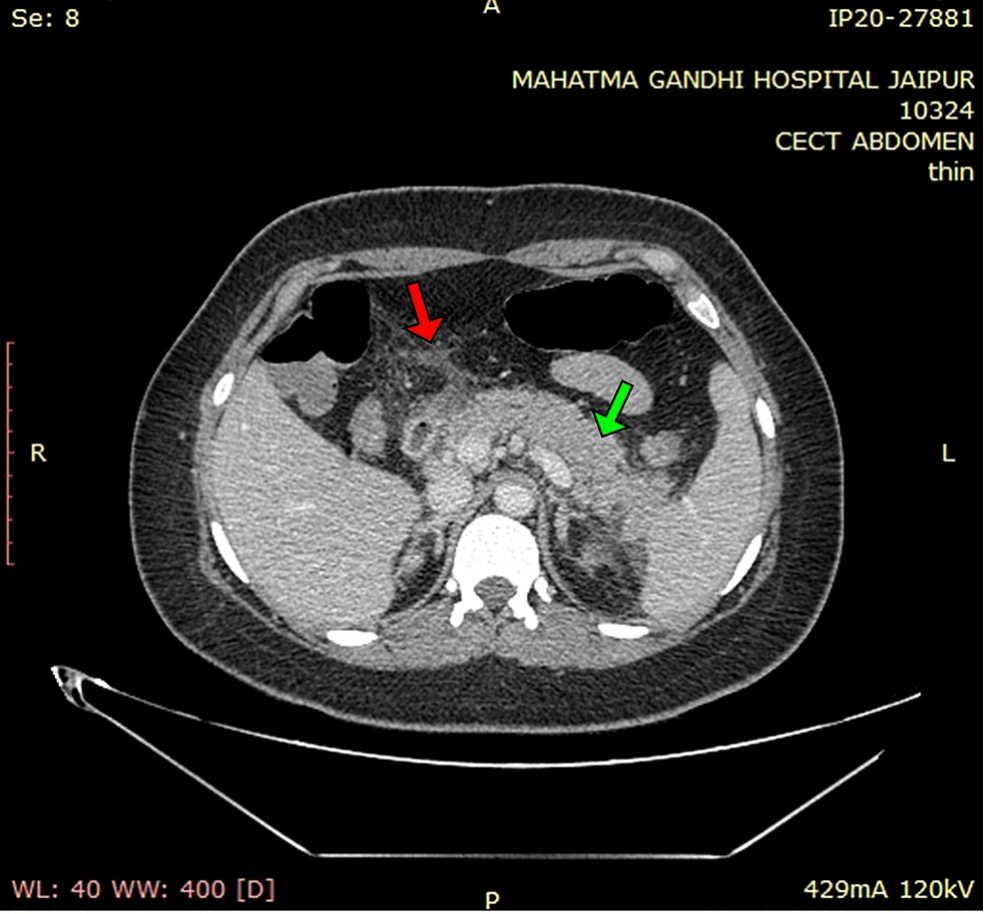
Contrast-enhanced CT of the abdomen (axial section) showing poorly enhancing, bulky pancreas, with no areas of necrosis (solid green arrow), pancreatic duct not dilated, and peri-pancreatic fat stranding (solid red arrow).

**Figure 3:**
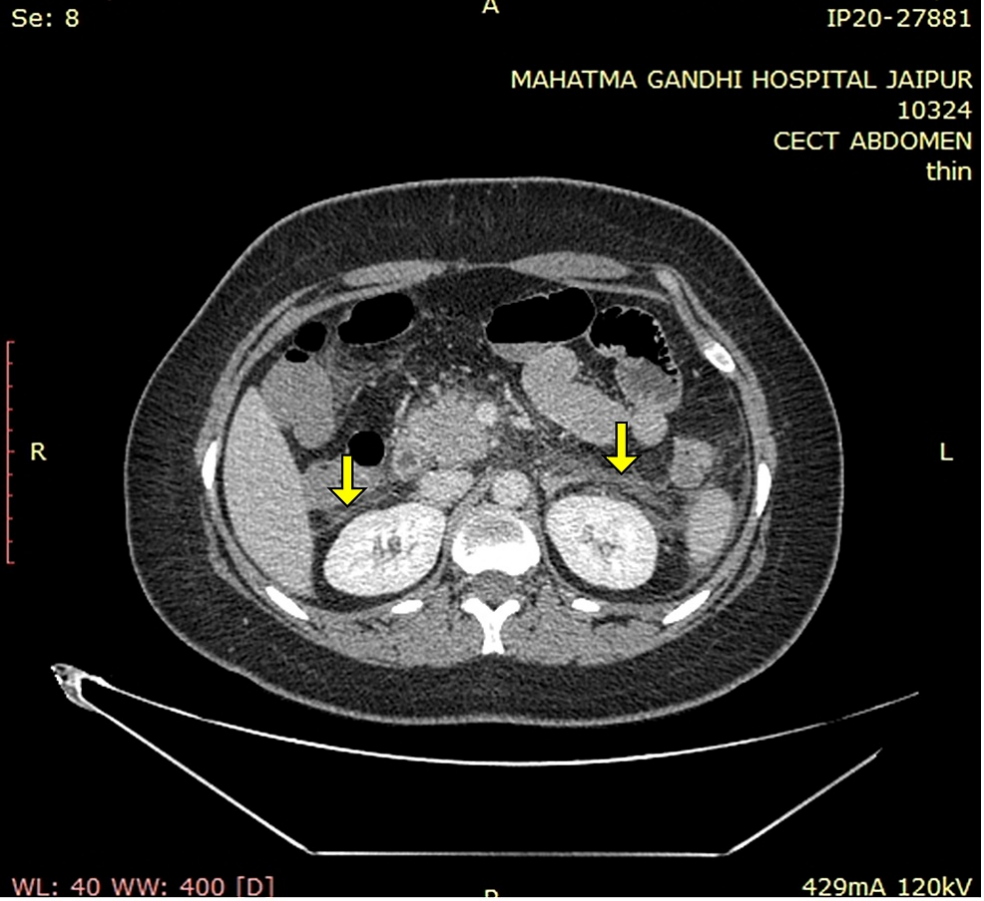
Contrast-enhanced CT of the abdomen (axial sections) showing bilateral renal and lateroconal fascial thickening (solid yellow arrows).

**Figure 4:**
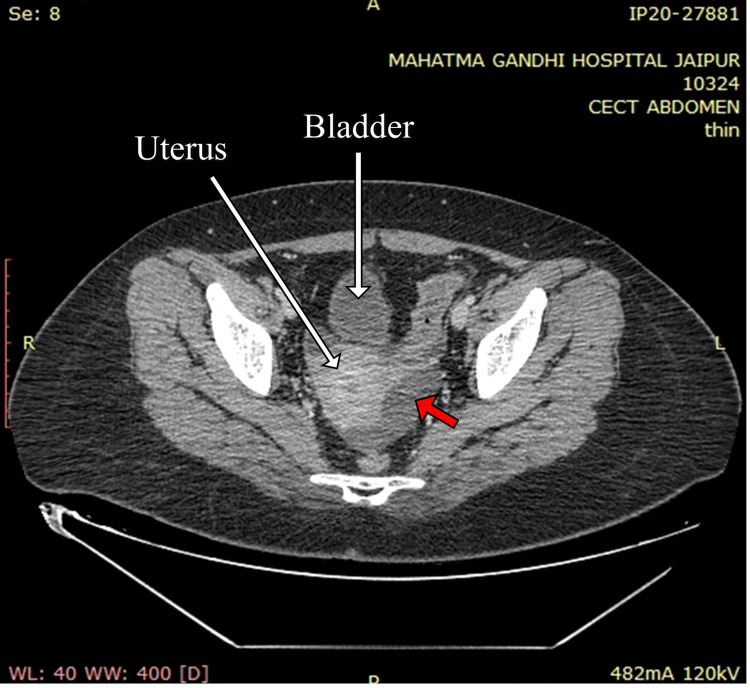
Contrast-enhanced CT of the abdomen (axial section) showing free fluid in the pelvis and ascites (solid red arrow).

A contrast-enhanced CT of the whole abdomen showed a bulky pancreas with poor enhancement, no parenchymal necrosis or calcification (Figures 1, 2), with soft tissue stranding and inflammatory changes in peripancreatic and mesenteric fat; renal and lateroconal fascial thickening (Figure 3); and mild free fluid in peritoneal cavity and pelvis (Figure 4); which further confirmed our diagnosis of acute interstitial pancreatitis (fulfilling all three criteria of acute pancreatitis as per the revised Atlanta classification) with mild ascites (modified CT Severity Index - 6).

**Figure 5:**
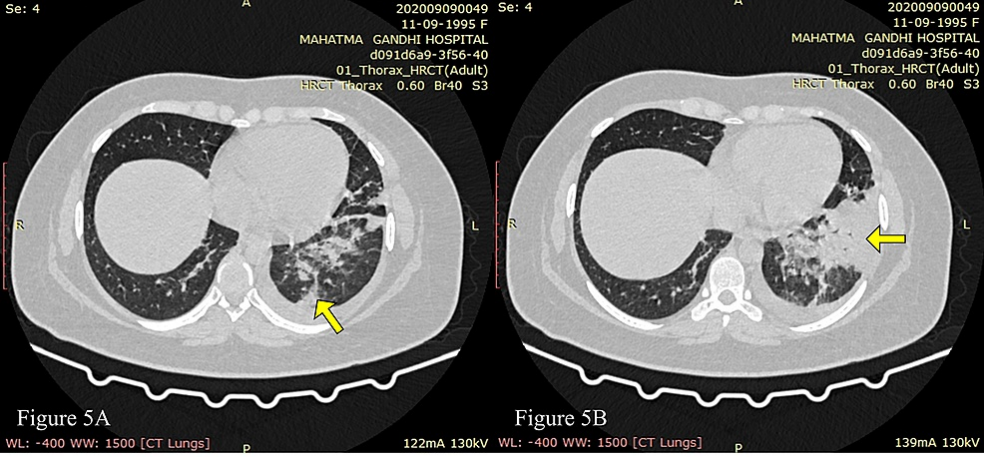
HRCT of the chest lung window (axial sections) showing ground-glass opacities and consolidation predominantly involving the left lower lobe of the lung (solid yellow arrows). CT severity score: 3/25. HRCT, high-resolution CT

**Figure 6:**
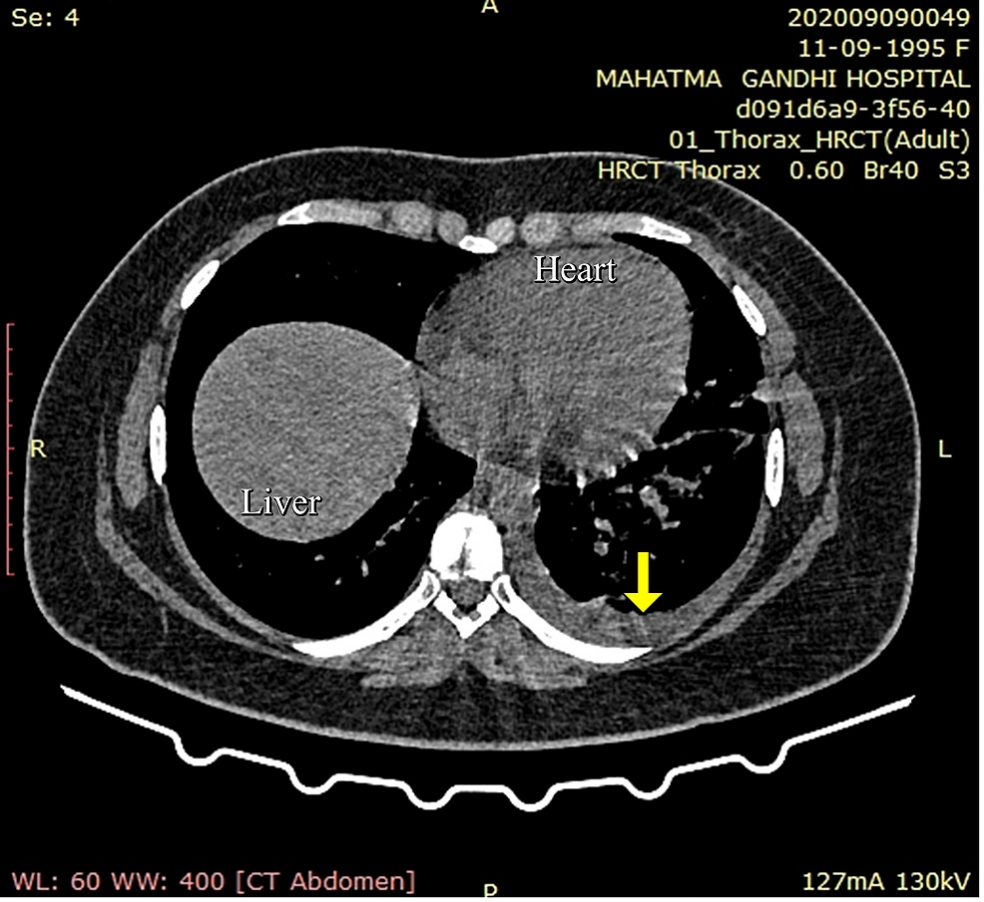
HRCT of the chest mediastinal window (axial section) showing left pleural effusion (solid yellow arrow). HRCT, high-resolution CT

The high-resolution CT of the chest showed patchy ground-glass opacities in the left lung, CO-RADS 6 ([COVID-19 Reporting and Data System] RT-PCR positive), CT severity score of 3/25 (Figures 5A, 5B), and bilateral pleural effusion (left > right) (Figure 6).

**Figure 7:**
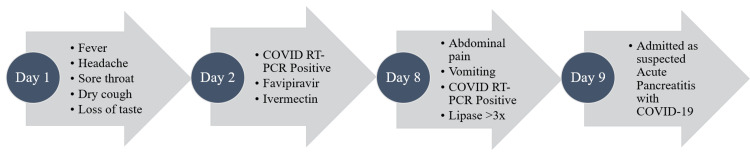
Timeline of symptoms: treatment initiated prior to admission.

**Figure 8:**
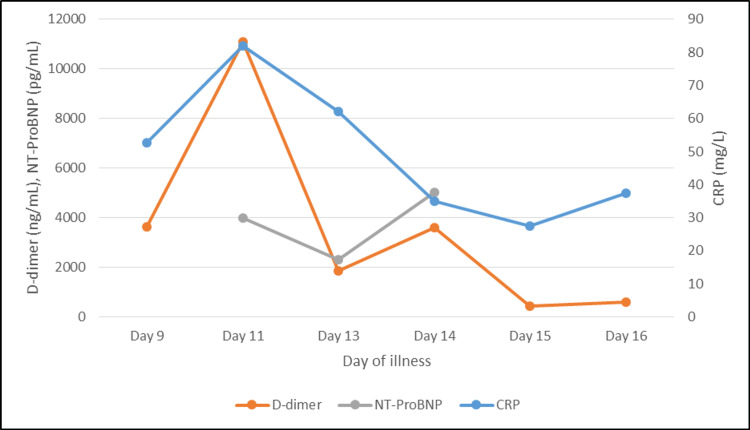
Graphical representation of CRP, D-dimer, and NT-proBNP values during the course of illness (after admission). CRP, C-reactive protein; NT-proBNP, N-terminal-pro-B-type natriuretic peptide

A screening 2D-ECHO (echocardiography) indicated no regional wall motion abnormality and a left ventricular ejection fraction of 60%. She was managed conservatively and kept nil per os with nasogastric suction, judicious intravenous (IV) fluids, IV antibiotics (piperacillin-tazobactam and metronidazole), IV methylprednisolone 40 mg once daily, subcutaneous enoxaparin 60 mg twice daily, and other supportive measures. On her third day of admission (day 11 of illness), she required transfer to the intensive care unit for respiratory distress and increased oxygen requirement. She was also switched to IV imipenem and started on IV remdesivir. The possibility of microthrombi formation in the pulmonary circulation (highly elevated D-dimer: 11,100 ng/mL), leading to pulmonary hypertension and myocardial stress (elevation of NT-proBNP [N-terminal-pro-B-type natriuretic peptide]: 3,990 pg/mL) was kept, and the patient was started on IV furosemide 20 mg once daily. The patient started showing significant improvement in abdominal pain and shortness of breath, and her oxygen requirement started decreasing. Her repeat COVID RT-PCR was negative on days 13 and 14 of illness (days 5 and 6 of admission), and she was able to tolerate oral liquid diet. She was off oxygen support and able to tolerate full oral diet by day 7 of admission and was discharged on day 9 of admission. A timeline of events (symptoms, treatment initiated) prior to admission is depicted (Figure 7). A graphical representation of her CRP (C-reactive protein), D-dimer, and NT-proBNP during the course of illness (after admission) is depicted (Figure 8).

## Discussion

Patients with COVID-19 frequently present with gastrointestinal symptoms. In a report of over 3,70,000 cases of COVID-19, 19% had diarrhea, 12% had nausea/vomiting, and 7.6% had abdominal pain [7]. A meta-analysis and systematic review of 60 studies of gastrointestinal manifestations of severe acute respiratory syndrome coronavirus 2 (SARS-CoV-2) infection estimated the pooled prevalence of gastrointestinal symptoms at 17.6% overall (13% for diarrhea, 10% for nausea/vomiting, and 9% for abdominal pain) [8], particularly higher in the severely ill. However, to date, there have not been many case reports of acute pancreatitis in COVID-19.

The diagnosis of acute pancreatitis requires two out of the following three features (as per the revised Atlanta classification) [9]: (1) abdominal pain consistent with acute pancreatitis, (2) serum lipase or amylase activity at least three times greater than the upper limit of normal, and (3) characteristic findings of acute pancreatitis on contrast-enhanced CT or MRI or USG. The common causes of pancreatitis, which were ruled out in this case, included biliary tract disease (e.g. gallstones), alcohol, hypertriglyceridemia, hypercalcemia, trauma, history of surgical procedures/post-ERCP (endoscopic retrograde cholangiopancreatography), developmental anomalies, and tumors. Drug-induced pancreatitis has not been reported with either ivermectin or favipiravir. Infections with viruses, helminths, and protozoa are known to cause pancreatitis, and viral infections such as those from hepatitis viruses, *Coxsackie* viruses, *Echoviruses*, mumps virus, cytomegalovirus, Epstein-Barr virus, *Varicella Zoster* virus, and influenza virus (H1N1) are the most common infectious cause (65% cases) of acute pancreatitis [10].

Anand et al. [11] reported the development of acute pancreatitis in a patient 10 days after the first positive PCR report. The patient was readmitted after discharge with fever, abdominal pain, and constipation, and CT obtained on day 3 of readmission indicated acute pancreatitis [11]. They raised the possibility of an association between this novel coronavirus and acute pancreatitis [11]. The temporal presentation of acute pancreatitis in their case matches that of our case; however, a serum amylase level test was not done in their patient on admission.

Another case report of acalculous acute pancreatitis in a COVID-19 patient was reported by Meireles et al. [12], where the patient developed nausea, vomiting, and pain abdomen on the 11th day of the disease. Her investigations revealed a 10-fold rise in her amylase and lipase levels, and imaging did not reveal any evidence of calculi or ischemia [12]. They suggested that pancreatic involvement likely arose from an immune inflammatory response to the virus (rather than direct viral invasion) given the temporal association between the development of pancreatitis and the clinical picture [12].

Hadi et al. [13] described the cases of three family members admitted with COVID-19, two of whom developed severe acute pancreatitis. The authors did not find a reason as to why only two of the five cases in the family developed acute pancreatitis. Both of them had severe pancreatitis with acute respiratory distress syndrome (ARDS) and multi-organ failure, and whether severe pancreatitis caused the ARDS and multi-organ failure or COVID-19 caused it could not be ascertained [13]. Kumaran et al. [14] reported the case of a 67-year-old hypertensive female who presented with one-day history of epigastric pain, nausea, and vomiting and was found to have acute necrotising pancreatitis after due investigations. In that case also, common causes of acute pancreatitis were ruled out, but the patient did have a positive history of superior mesenteric artery stenosis one year ago, for which she underwent surgery and was on apixaban. She later developed tachypnea and hypoxia and was found to be COVID-19 positive [14]. It highlights the fact that patients with COVID-19 may even present with pancreatitis, and the worsening of respiratory system after the development of acute pancreatitis in their case matches that of our case.

The presence of viral RNA in fecal specimens and gastrointestinal epithelium despite negative respiratory tests has led to the suggestion of a possible feco-oral transmission route for SARS-CoV-2 [15]. The expression of ACE-2 ([angiotensin-converting enzyme 2] the host receptor for SARS-CoV-2 entry) is very high in the pancreas and gastrointestinal epithelium [16].

The endocrine part of the pancreas strongly expresses ACE-2, whereas exocrine tissues are only weakly positive; consequently, there are very few reports of pancreatitis in patients with SARS [17] or COVID-19. Wang et al. [18] reported that 17% patients of COVID-19 had evidence of pancreatic injury (defined as elevation of pancreatic enzymes), and two-thirds of those patients had abnormal blood glucose levels [18]. They attributed this injury possibly due to either direct cytopathic effect of SARS-CoV-2 or mediated by the immune response directed against SARS-CoV-2 [18]. In another study by Liu et al. [19] of 121 patients with COVID-19, only one out of 54 mild cases had pancreatic enzyme elevation, but in severe cases, 12 out of 64 cases (17.9%) had enzyme elevation, but no case had necrotising pancreatitis [19]. Clinicians should have a high index of suspicion for pancreatic injury in patients with COVID-19 (especially in those with pancreatic enzyme elevation), as it can be a life-threatening complication.

There are some lessons to be learned here from the 2002-2004 SARS epidemic. The pancreatic involvement in SARS was not only limited to pancreatic enzyme elevation. The development of acute diabetes in 20 out of 39 patients of SARS (who were initially non-diabetic) was noted by Yang et al. [17]. Of those 20 patients, 18 returned to normoglycemia in the three-year follow-up of the study [17]. This indicated that SARS-CoV-1 caused damage to pancreatic islets and caused acute diabetes. Considering the genetic similarities between SARS-CoV-1 and 2, the potential for permanent pancreatic damage, chronic pancreatitis, and acute or chronic diabetes due to SARS-CoV-2 cannot be overlooked.

Hanley et al. [20] reported the histopathological post-mortem findings in 10 patients over the age of 18 years who died due to COVID-19. The most consistent findings were diffuse alveolar damage, thrombosis, and immune cell depletion primarily affecting the lungs, hearts, and kidneys [20]. But the unexpected finding was pancreatitis in two patients, with the first having gross necrotising hemorrhagic pancreatitis and the second having only microscopic evidence of acute inflammation in the pancreas [20]. While it was not obvious what determines the spread of SARS-CoV-2 outside the respiratory tract (as the sites of highest ACE-2 expression did not correlate with severity of pathological involvement), it possibly depends on several other factors such as cellular receptor status, route of transmission, and host factors (genetic makeup, TMPRSS2 expression/polymorphism, etc.) [20].

## Conclusions

Respiratory, cardiac, renal, and multi-organ failure, as well as thrombosis are the most common causes of mortality in patients with COVID-19. Acute pancreatitis can be a rare cause of mortality, especially in those with moderate-to-severe disease. Patients with COVID-19 may be screened for pancreatic injury, as it may have an effect on prognosis. Larger studies are required to confirm the cause and pathogenesis of acute pancreatitis in COVID-19.
